# Sex Difference in Cigarette-Smoking Status and Its Association with Brain Volumes Using Large-Scale Community-Representative Data

**DOI:** 10.3390/brainsci13081164

**Published:** 2023-08-04

**Authors:** Xiaofei Chen, Riley Cook, Francesca M. Filbey, Hang Nguyen, Roderick McColl, Haekyung Jeon-Slaughter

**Affiliations:** 1 Department of Statistics and Data Science, Southern Methodist University, Dallas, TX 75205, USA; xiaofeic@mail.smu.edu (X.C.); hangnguyen@mail.smu.edu (H.N.); 2 VA North Texas Health Care Service, Dallas, TX 75216, USA; riley.cook@va.gov; 3 School of Behavioral and Brain Sciences, University of Texas at Dallas, Richardson, TX 75080, USA; francesca.filbey@utdallas.edu; 4 Department of Radiology, University of Texas Southwestern Medical Center, Dallas, TX 75390, USA; roderick.mccoll@utsouthwestern.edu; 5 Department of Internal Medicine, University of Texas Southwestern Medical Center, Dallas, TX 75390, USA

**Keywords:** cigarette smoking, brain volumes, sex, subcortical region, lateral ventricle

## Abstract

Background: Cigarette smoking is believed to accelerate age-related neurodegeneration. Despite significant sex differences in both smoking behaviors and brain structures, the active literature is equivocal in parsing out a sex difference in smoking-associated brain structural changes. Objective: The current study examined subcortical and lateral ventricle gray matter (GM) volume differences among smokers, active, past, and never-smokers, stratified by sex. Methods: The current study data included 1959 Dallas Heart Study (DHS) participants with valid brain imaging data. Stratified by gender, multiple-group comparisons of three cigarette-smoking groups were conducted to test whether there is any cigarette-smoking group differences in GM volumes of the selected regions of interest (ROIs). Results: The largest subcortical GM volumetric loss and enlargement of the lateral ventricle were observed among past smokers for both females and males. However, these observed group differences in GM volumetric changes were statistically significant only among males after adjusting for age and intracranial volumes. Conclusions: The study findings suggest a sex difference in lifetime-smoking-associated GM volumetric changes, even after controlling for aging and intracranial volumes.

## 1. Introduction

According to the Centers for Disease Control and Prevention (CDC), cigarette-smoking-related health problems account for about one in every five deaths in the United States each year and are the leading preventable cause of death [1]. However, relapses are common despite treatment, with cigarette-smoking cessation interventions showing limited success overall [2,3]. Within addiction research, the failure of smoking cessation has been mostly attributed to nicotine dependence [4,5] and its influence on a wide variety of cognitive domains [6], such as sensory processing [7], executive functioning [8], and memory [9], among others. In particular, subcortical regions of the brain have been implicated by research on substance abuse and dependence. For instance, subcortical regional volume recovery was found to be associated with the absence of alcohol or cocaine [10], while decreased subcortical and striatal volumetric changes were associated with the abuse of and dependence on other substances, such as cocaine [11], alcohol [12], and other stimulants [10,13].

Biological sex is a putative factor for brain structural changes with nicotine use, given that male brain volumes are larger to start with and are more susceptible to volumetric loss with aging than female brains [14]. Also, cigarette-smoking behaviors differ by sex [15]. Therefore, sex stratification is suitable for examining cigarette-smoking associations with subcortical brain structural changes [16].

Sex may play a major role in brain structural changes related to cigarette smoking [16], as several environmental, sociocultural, and biological factors have been shown to mediate substance use and its effects on the brain [17]. Overall, lower GM volumes in multiple subcortical regions of the brain in chronic smokers across sexes have been reported [18]; however, the relations between sex, smoking status, and volumetric deficits require further evaluation.

The existing literature reports significant relationships between cigarette smoking and decreased gray matter (GM) volumes in subcortical regions of the parahippocampus, thalamus, pallidum, and amygdala, as well as connected structures such as the insula [18,19,20,21,22]. However, the current literature is equivocal in parsing out a normal aging-related volume reduction and sex differences in cigarette smoking related to GM volume reduction. This could be partly due to the small scale and non-representativeness of previous data.

Epidemiological studies have reported GM volume differences between active or ever-smokers and never-smokers [23,24,25] but have failed to discriminate significant GM volume differences among past smokers who were able to quit smoking during their lifetime from either active or never-smokers [24]. However, some researchers reported that quitting smoking was positively associated with larger GM volumes [26]. Some studies have demonstrated that cessation might recover the loss of GM volumes associated with cigarette smoking [27,28]. However, the generalization of these results is limited due to the samples being small in size and not representative of the adult population. A large, representative study of the adult population is needed to further investigate whether quitting cigarette smoking is associated with larger GM volumes.

To address this, the current study employed a post-stratification method, stratified by sex, using large-scale, community-representative data from the Dallas Heart Study (DHS). The current study examined subcortical and lateral ventricle brain region GM volume differences between two smoking groups—active and past smokers—and those who have never smoked (never-smokers), stratified by sex.

## 2. Materials and Methods

The current study extracted data from 3278 Dallas Heart Study (DHS) participants who were English- or Spanish-speaking, aged 18 years or older, and residing in Dallas County [29]. The DHS is a large, multi-ethnic, probability-based population sample of adult English- or Spanish-speaking Dallas County residents, with intentional oversampling of African Americans to make up 50% of the original study participants [29]. The DHS’s primary goal was to collect data via health surveys and state-of-the-art imaging studies and improve the diagnosis, prevention, and treatment of cardiovascular disease (further information is available at https://www.utsouthwestern.edu/edumedia/edufiles/research/center_translational_medicine/dallas_heart_study/dhs-study-overview.pdf; accessed on 1 January 2023). The study was conducted from September 2007 to December 2009 and collected data on demographics, disease conditions, and history of cigarette smoking and alcohol consumption, as well as brain imaging data. A total of 2087 DHS participants underwent brain imaging acquisition, with a final ethnic composition of 45.7% African American, 35.1% White, 17.1% Hispanic, and 2.1% other race [30]. The final sample size of the study was 1959 participants with valid volumetric quantification data.

### 2.1. Demographic and History of Smoking and Alcohol Use Data

The demographic characteristics included are gender, age, racial/ethnic background, Body Mass Index (BMI, kg/m^2^), and annual family income levels in US dollars.

History of cigarette smoking (smokers were defined as having smoked more than 100 cigarettes in their lifetime) was used as a variable to create three smoking groups—active, past, and never-smokers. Active smokers were defined as those with self-reported cigarette consumption in the past 2 weeks. Past smokers were defined as those who have smoked more than 100 cigarettes throughout their lifetime but have not smoked in the last 6 months. The never-smokers were defined as those who have smoked 100 or fewer cigarettes in their lifetime. In addition, the duration of active cigarette smoking was measured in years for both active and past smokers.

Alcohol use was measured by quantity (i.e., ‘‘During this phase of your drinking, how many drinks did you usually have at one time?’’, where one drink is equivalent to one 12 oz (354.88 mL) can of beer, one 4 oz (118.29 mL) glass of wine, or one 1 oz (29.57 mL) shot of liquor) and frequency measures (i.e., ‘‘In the past 12 months, how often did you drink any kind of alcoholic beverage?’’). Multiplying the quantity and frequency scores provided a quantity–frequency index (QFI), which reflects an individual’s average number of drinks per year in the past 12 months [31].

### 2.2. Brain Imaging Data

Whole-brain, 2-dimensional, fluid-attenuated inversion recovery and 3-dimensional, magnetization-prepared, rapid acquisition with gradient echo Magnetic Resonance (MR) imaging at 3.0 T was performed for Dallas Heart Study participants who consented to MR imaging [32,33,34]. Quantification of MR imaging was performed using two freely available neuroimaging software packages: Functional MR Imaging of the Brain Software Library, available at http://fsl.fmrib.ox.ac.uk/fsl/fslwiki accessed on 1 January 2023 [35,36], and FreeSurfer, available at https://surfer.nmr.mgh.harvard.edu accessed on 1 January 2023. The functional MRI method of auto-segmentation of brain regions is described elsewhere [37].

### 2.3. Regions of Interests (ROIs)

Cross-sectional smoking status differences in gray matter (GM) volume structure among normal healthy DHS study participants were investigated in subcortical brain regions [34], as well as in insular and lateral ventricle regions. Thus, the selected regions of interest (ROIs) are the amygdala, insula, hippocampus, thalamus, caudate, putamen, pallidum, nucleus accumbens, and lateral ventricle, all of which were implicated in cigarette smoking in the previous literature. The left and right GM volumes of ROIs were measured separately and extracted from the DHS brain imaging data. Total GM volumes were calculated by summing up the left and ride sides of GM volumes per ROI.

### 2.4. Statistical Methods

Descriptive statistics were used to examine the three smoking groups’ differences in the demographic and clinical characteristics of the study participants. The Chi-square test and t-statistics were used to test smoking group differences in categorical and continuous variables, respectively. Furthermore, |standard residual| > 3 was set to test the true associations of smoking groups with categorical variables. The study employed a post-stratification method [38], stratified by sex, to precisely estimate smoking group differences in GM volumes. The generalized linear model was used to calculate each study respondent’s adjusted GM volumes for age and intracranial volume. Bar plots are used to display means and standard deviations of GM volumes of ROIs per smoking group, stratified by gender (R packages *ggpubr* and *rstatix*). In addition to the original GM volumes from the data (unadjusted), the study also adjusted GM volumes by age and intracranial volumes (age- and intracranial-volume-adjusted GM volumes) by running a generalized linear model with gamma transformation (SAS 9.4 Proc Glimmix). All statistical and graphic analyses were conducted using SAS 9.4 (SAS Institute, Cary, NC, USA) and the R statistical program, 4.1.3 version (cran-r.project.org).

Multiple-group comparisons of the three cigarette-smoking groups were conducted to test whether there were any cigarette-smoking group differences in either unadjusted or adjusted GM volumes following the Hochberg approach (1988) [39] to control the Family-Wise Error Rate (FWER, type-I error α at 0.05) for multiple comparisons (R function p. adjust(method = ’hochberg’)). Pearson correlation was used to examine correlations of GM volumes with continuous covariates, including age, income, and alcohol consumption levels.

## 3. Results

The study data included 1160 females and 799 males. Among females, 18.4% (*n* = 214) were active cigarette smokers, 19.6% were past smokers (*n* = 227), and 62.0% were never-smokers (*n* = 719). Among males, 23.5% (*n* = 214) were active cigarette smokers, 28.0% were past smokers (*n* = 224), and 48.4% were never-smokers (*n* = 387). Table 1 depicts descriptive statistics by smoking status, stratified by sex.

The past smokers were considerably older than both active and never-smokers for both females and males (about 4 years on average), while ages were similar between active and never-smokers for both sexes. On average, the family income level was the lowest among the active smokers and the highest among the never-smokers. Active smokers consumed significantly more alcohol than both past and never-smokers across genders, while alcohol consumption levels between past and never-smokers did not significantly differ. BMI levels in active smokers were significantly lower than both past and never-smokers for both sexes.

### 3.1. Smoking Status Group Comparisons: Unadjusted GM Volumes

#### 3.1.1. Active vs. Past Smokers in Unadjusted GM Volumes

**Figure 1:**
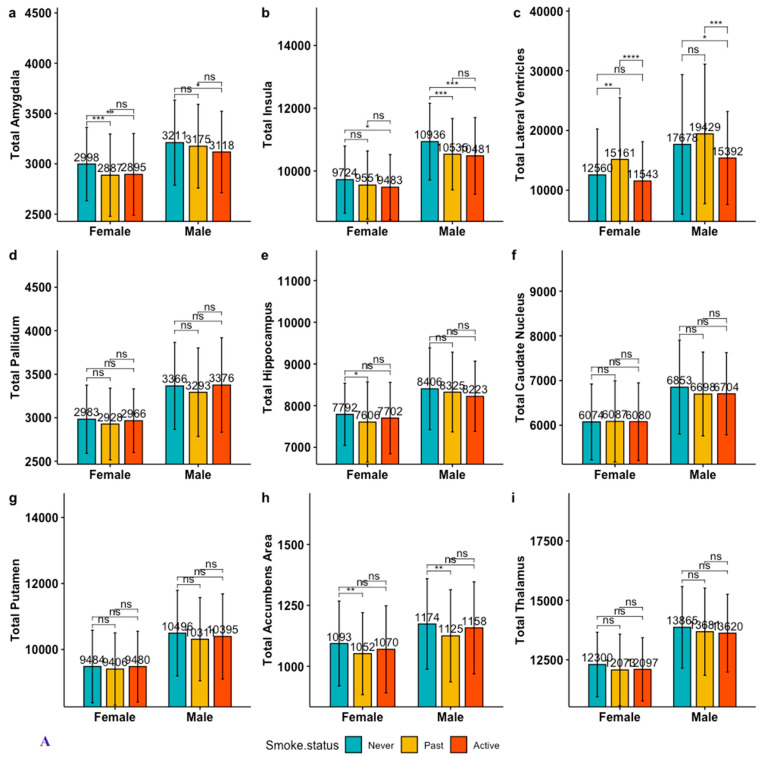

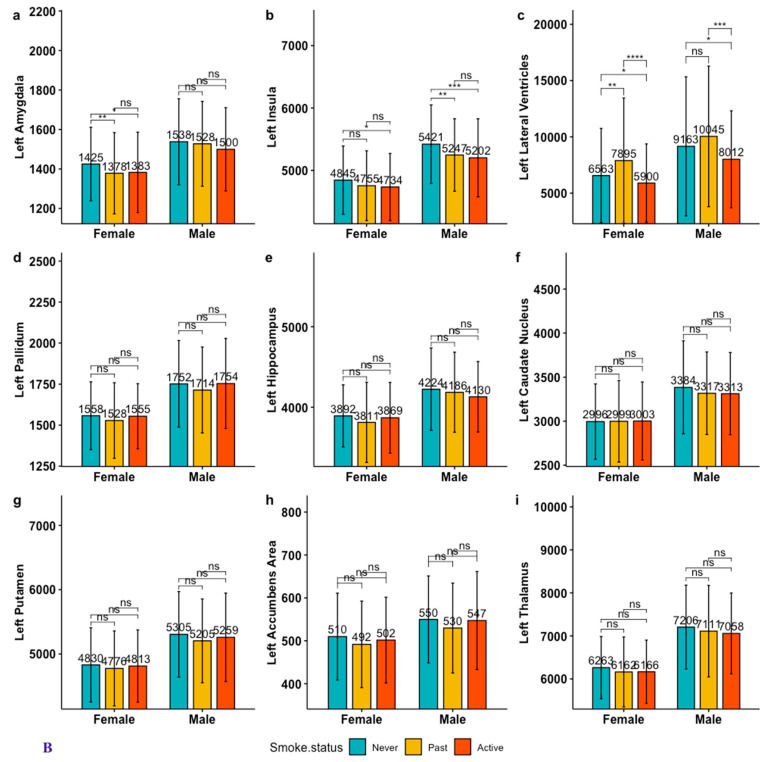

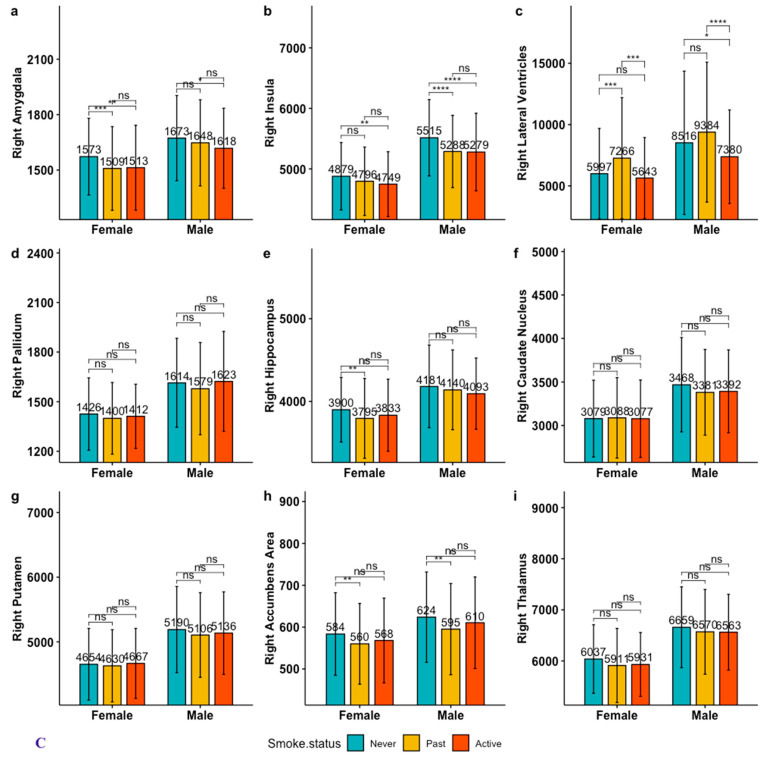
Pairwise comparison of unadjusted gray matter (GM) volumes among smoking groups stratified by sex. Notes. 1. Panel 1. (**A**). Total GM volumes, Panel 1. (**B**). Left-side volumes, Panel 1. (**C**). Right-side volumes; 2. GM volumes were compared pairwise between smoking groups: never-smokers vs. past smokers; never-smokers vs. active smokers; and past vs. active smokers. Hochberg correction was used for multiple-group comparison, statistical significance at α = 0.05; 3. ****: <0.0001; ***: <0.001; **: <0.01; *: <0.05; “ns”: not significant.

Among males and females, active smokers did not significantly differ in unadjusted GM volumes from past smokers in any of the ROIs, except for the lateral ventricle region. Active smokers’ unadjusted lateral ventricle volumes were significantly smaller than those in past smokers for both females and males (total, left, and right, *p* < 0.001; Figure 1A–C; also see Supporting Information Table S1).

#### 3.1.2. Active vs. Never-Smokers in Unadjusted GM Volumes

No significant group differences between active and never-smokers were observed in any of the hippocampal, palladium, caudate, putamen, nucleus accumbens, and thalamus volumes in either sex (Figure 1A–C). After multiple-comparison correction, unadjusted total and right amygdala and insular volumes in both female and male active smokers were smaller than those in never-smokers (Figure 1A,C). The left amygdalas in female active smokers were smaller than those in female never-smokers, while left insular volumes in male active smokers were smaller than those in male never-smokers (Figure 1B). Total and right lateral ventricle volumes in male active smokers were smaller than those in male never-smokers, while left lateral ventricle volumes were smaller in active smokers than those in never-smokers in both sexes (Figure 1A,C).

#### 3.1.3. Past vs. Never-Smokers in Unadjusted GM Volumes

Female past smokers showed significantly more deficits in unadjusted total and right GM volumes of the regions of the amygdala and hippocampus than female never-smokers, while they were similar in male groups. Only female past smokers presented with a significantly enlarged lateral ventricle area compared to their never-smoker counterparts (total *p* = 0.001, Figure 1A; left *p* = 0.002, Figure 1B; right *p* = 0.0001, Figure 1C), while lateral ventricle GM volumes were similar between male past and never-smokers (total *p* = 0.075, Figure 1C; left *p* = 0.092, Figure 1B; right *p* = 0.073, Figure 1C).

Past smokers’ unadjusted nucleus accumbens volumes were significantly smaller than those in the never-smokers for both sexes (Figure 1A–C). Male past smokers’ insular volumes were smaller than those in male never-smokers (total *p* = 0.0001, Figure 1A; left *p* = 0.001, Figure 1B; right *p* < 0.0001, Figure 1C), while they were similar among females (total *p* = 0.075; Figure 1A; left *p* = 0.069, Figure 1B; right *p* = 0.11, Figure 1C).

### 3.2. Smoking Status Group Comparisons: Adjusted GM Volumes for Age and Intracranial Volume

#### 3.2.1. Active vs. Past Smokers in Adjusted GM Volumes

**Figure 2:**
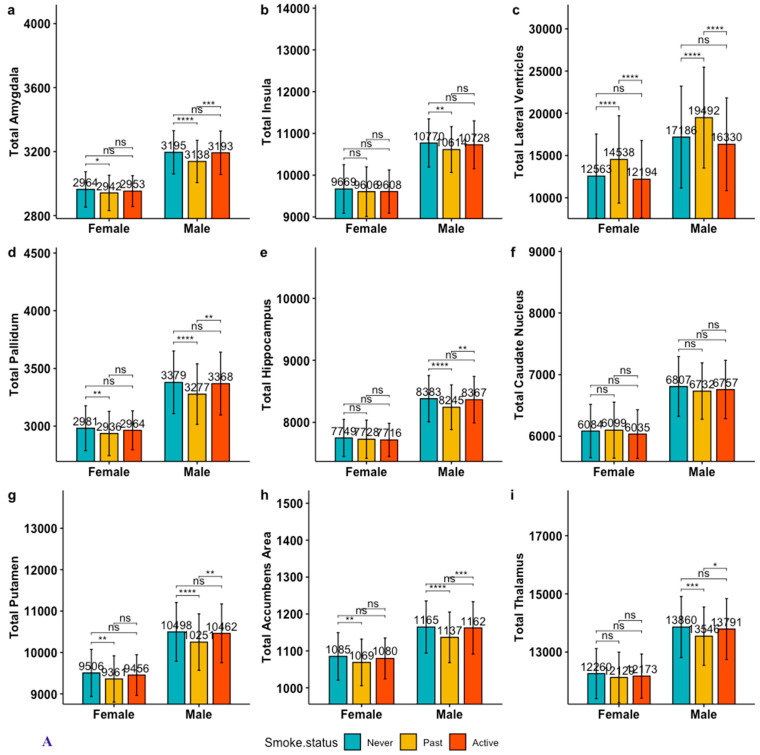

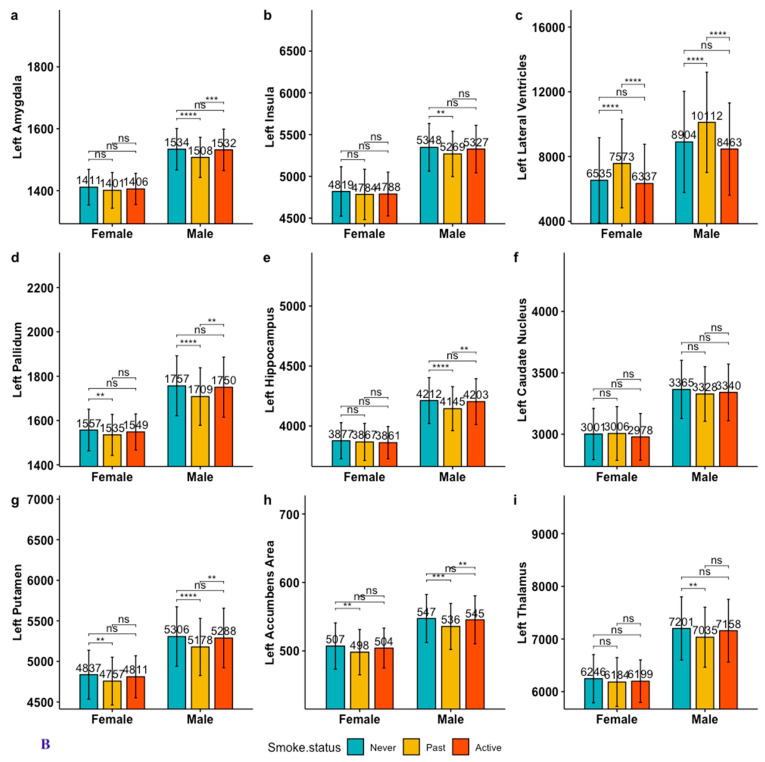

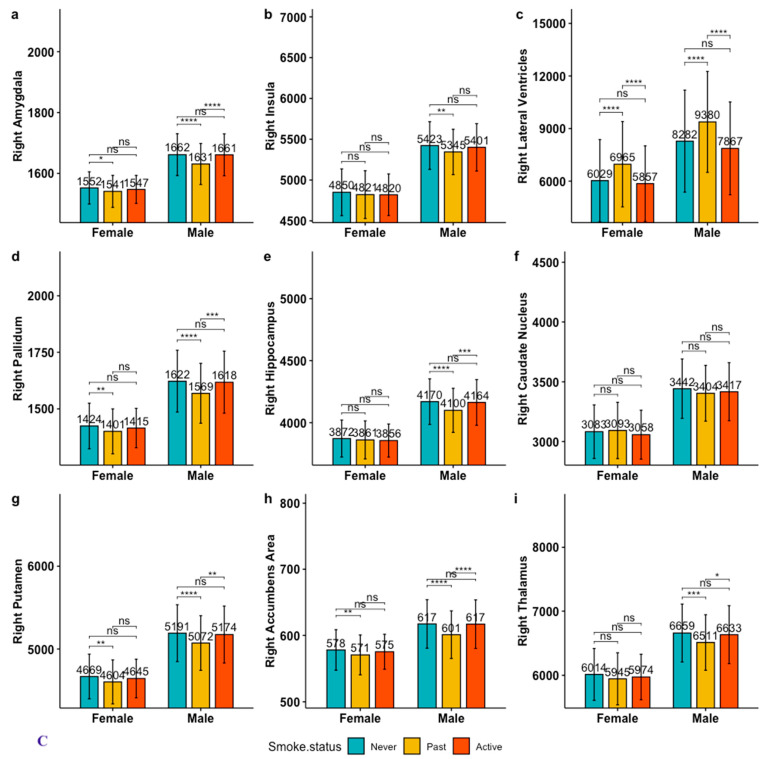
Pairwise comparison of adjusted gray matter (GM) volumes among smoking groups stratified by sex. Notes. 1. Panel 2. (**A**). Total GM volumes, Panel 2. (**B**). Left-side volumes, Panel 2. (**C**). Right-side volumes; 2. Age- and intracranial-volume-adjusted GM volumes were calculated by running a general linear model on GM volumes with age and intracranial volumes as covariates and saving as predicted GM volumes for each subject. These adjusted GM volumes were then used for smoking group comparisons, never-smokers vs. past smokers; never-smokers vs. active smokers; and past vs. active smokers. Hochberg correction was used for multiple-group comparison, statistical significance at α = 0.05; 3. ****: <0.0001; ***: <0.001; **: <0.01; *: <0.05; “ns”: not significant.

The group difference in adjusted GM volumes in the lateral ventricle region between active and past smokers was preserved for both sexes (Figure 2). Significantly larger adjusted GM volumes in the amygdala, hippocampus, putamen, pallidum, nucleus accumbens, and thalamus areas among active smokers compared to their past-smoker counterparts were observed among males only (Figure 2; also see Supporting Information Table S2), while the results were similar among females.

#### 3.2.2. Active vs. Never-Smokers in Adjusted GM Volumes

When considering age- and intracranial-volume-adjusted GM volumes, there were no significant GM volumes found in any of the ROIs between active and never-smokers for either sex (Figure 2).

#### 3.2.3. Past vs. Never-Smokers in Adjusted GM Volumes

After adjusting for age and intracranial volumes, sex differences in total amygdala volumes were no longer observed. While sex differences were observed in total insular and hippocampal volumes after adjusting for age and intracranial volumes, the findings were reversed. Female total insular and hippocampal volumes were similar between past and never-smokers after adjusting for age and intracranial volumes, while male past smokers’ adjusted insular and hippocampal regions showed significant total GM volumetric deficits compared to their never-smoker counterparts.

The statistical significance of larger deficits among the female past cigarette smokers compared to the female never-smokers was attenuated in the right-side amygdala and hippocampus in age- and intracranial-volume-adjusted GM volumes (Figure 2). On the other hand, significant deficits in adjusted GM volumes in male past smokers compared to their never-smoker counterparts were observed in both the left and right sides of all ROIs of interest, except for the caudate region (Figure 2).

For both females and males, past smokers had significantly enlarged lateral ventricle regions compared to never-smokers in adjusted total, left, and right GM volumes (Figure 2).

### 3.3. Aging-Related Subcortical Region Volumetric Deficits

Overall, this study found significant aging-related volumetric deficits in subcortical structures, except for lateral ventricle regions, in both left- and right-side as well as total GM volumes (*p* < 0.01). Larger lateral ventricle GM volumes were positively associated with older age for both genders (*p* < 0.01).

### 3.4. Alcohol Consumption and Subcortical Region Volumetric Deficits

The quantity of alcohol consumption was positively correlated with total (*p* = 0.045), left (*p* = 0.046), and right thalamus volume losses (*p* = 0.046) and right hippocampus loss (*p* = 0.018) among males only, while no female brain ROI volumes were significantly correlated with alcohol consumption.

## 4. Discussion

This study applied a post-stratification method, stratifying by sex, to a large-scale, community-population-representative brain imaging data set to evaluate the relations between cigarette-smoking status and GM volumetric changes in subcortical and lateral ventricle brain regions. In contrast to the traditional Analysis of Variance (ANOVA) method of estimating an interaction term between sex and smoking groups, post-stratification is a method often used to increase the precision of estimation when the variable was not considered in the original study sampling. Post-stratification, stratifying by sex, would increase the precision of estimating smoking effects on GM volumes if there were large sex differences in brain GM volumes, which were not controlled for.

Overall, the largest subcortical GM volumetric loss was observed among past smokers for both females and males. However, the significantly larger subcortical brain region GM volumetric deficits and enlargement of the lateral ventricle among the past smokers compared to the never-smokers were observed only among males, not females, after adjusting for age and intracranial volumes. There was a significant sex difference in adjusted GM volumes in ROIs of the insula, hippocampus, and thalamus between past and never-smokers—significantly smaller in the male past smokers compared to the male never-smokers but similar between the female past and never-smokers.

Adjusted GM volumes in all ROIs, except for the insula and caudate, among the past smokers were significantly smaller than those among the active smokers for males only. The past smokers in our study data were significantly older and had larger intracranial volumes than their active- and never-smoker counterparts for both sexes (Table 1). While significant GM volumetric deficits in the amygdala and insula among active smokers compared to the never-smokers were observed for both sexes, the statistical significance disappeared after adjusting for age and intracranial volumes. No sex differences were observed in adjusted GM volumes between active and never-smokers in any of the ROIs of interest.

Within the context of healthy aging, it is recognized that the GM volumes of various subcortical brain regions [40] decrease with aging, as well as other brain regions, including the frontal region [41,42]. Conversely, the enlargement of the lateral ventricle region has been noted as a biomarker for the healthy aging process [43,44]. Consistent with previous research, this study observed a cigarette-smoking association with accelerated age-related volume losses in several subcortical brain regions [25]; however, this may also be confounded by chronic diseases and alcohol consumption.

In unadjusted GM volumes, we observed significant GM volume deficits among female active smokers compared to their never-smoker counterparts on both the left and right sides of the amygdala and insular regions as well as the right sides of the hippocampus, nucleus accumbens, and thalamus. However, the female sex’s amplifying effect on smoking-related volumetric deficits in these ROIs dissipated after adjusting for age and intracranial volumes. This suggests that the observed subcortical GM volume deficits among female active smokers may not be associated with cigarette-smoking behaviors but rather are due to healthy aging and individual differences in cranial sizes.

Cigarette smoking is commonly prevalent among patients with chronic diseases, where some studies have reported significant brain GM volumetric losses among individuals with diagnoses such as cardiovascular disease, COPD [45], Type II diabetes [46], and schizophrenia [34]. The use of nicotine may play several roles in relation to disease, such as a causal factor contributing to illness [47] or as an agent that may interact with pathophysiology [48]. Unlike the previous literature based on small clinical and disease samples, the current study findings are from large-scale, healthy community sample data. Thus, the observed association between cigarette smoking and subcortical GM volumetric deficits among healthy populations is less likely to be confounded by chronic diseases.

Similarly, alcohol consumption is an established factor responsible for accelerating brain GM volume loss. Research has shown significant adverse health effects of synergistic alcohol intake and cigarette smoking on neurocognition and neurobiology [49,50]. High comorbidity rates have been observed between alcohol and nicotine use, with studies showing that an individual is three times more likely to be a smoker if they are dependent on alcohol, while those who are dependent on nicotine are four times more likely to be dependent on alcohol [15,51,52]. This was similarly reflected in the results of our study, where alcohol drinking was highly prevalent among smokers, and its consumption level was significantly higher among active smokers compared to past and never-smokers. Overall, 14% of subjects had missing data on annual alcohol consumption (15% in females, with 17% in female never-smokers; 12% in males) and, thus, were not included in the main analyses. The study conducted a further analysis to examine smoking group differences in alcohol-consumption-adjusted GM volumes using only subjects with complete data on alcohol consumption (see Supporting Information Table S3 and Figure S1). Additional analyses showed that after further adjusting for alcohol consumption, the largest subcortical GM volumetric loss among past smokers was observed only in male brains, not females. However, the female active smokers’ subcortical GM volumes were significantly smaller than the female never-smokers’, while similar GM volumes were found between male active and never-smokers. Considering that there were substantial missing data on alcohol consumption in female never-smokers and no significant association between the alcohol consumption level and volume deficits in ROIs of females’ brains, a future study is warranted to examine whether there is a true sex difference in alcohol confounding effects on cigarette-smoking-associated GM volumetric loss.

Some studies have reported associations between the failure of smoking cessation and the loss of GM volumes [27], with the implication being that subcortical brain region GM volumes could serve as a marker for predicting the success of smoking cessation. Contrary to the existing literature, this study found that active smokers’ subcortical brain region GM volumes were similar to the never-smokers’ and consistently larger than past smokers’ for both sexes after adjusting for age and intracranial volumes. The study finding of a significantly larger GM volumetric loss among only males who were able to quit smoking during their lifetime suggests that the positive impact of quitting smoking on the recovery of cigarette-smoking-related brain volumetric loss may vary by sex and among individuals, in contrast to the previous literature [26,27]. However, these findings may be specific to our data. The past smokers in our study were significantly older than both active and never-smokers across sexes, with an average of 35 smoking years during their lifetime before cessation, which was, on average, 4 years longer than the active smokers. With this, smoking cessation may have been delayed for too long, thus making it too late for past smokers to recover the loss of GM volumes associated with cigarette smoking despite quitting smoking. At the same time, the active smokers in our study were healthy community samples whose health conditions did not force smoking cessation, whereas, typically, many smokers quit due to deteriorating health conditions. Also, it is noted that the existing research identifying ROIs associated with smoking history and cessation has been inconsistent overall. As extensive brain imaging and longitudinal data can be difficult to capture in large quantities, much of the existing literature has been based on small sample sizes, yielding mixed results.

Several studies have evaluated the relationships of cigarette smoking with other issues of public health, such as income levels and body weight, among others. Consistent with the previous literature, the family income level was the lowest in active smokers and the highest in never-smokers in our study. Previous research has shown that the role of income in smoking behavior is closely related to the propensity to smoke [53]. Further, the price of cigarettes has been found to hold the greatest participation effects found among low-income populations, particularly women, while higher family earnings reduced participation for both men and women and reduced consumption for women [53]. Additionally, BMI levels have been associated with cigarette smoking, in which duration and cessation have both been shown to play a role in body-weight changes [54]. Our study finding that BMI levels were lower in active smokers than in both past and never-smokers is also consistent with the existing literature.

We conducted an additional analysis adjusting for the duration of smoking and found similar results to age- and intracranial-volume-adjusted GM volumes between active and past smokers (Table S4).

Although our study benefits from a large, representative sample, limitations are noted. First, a substantial portion of the study sample had missing data on alcohol consumption, with a prevalence in females and never-smokers. Therefore, the main analysis omitted alcohol consumption as a factor that mitigates the cigarette-smoking relation w subcortical brain region structural changes due to a potential bias from non-random missing data. Second, the study data on smoking behaviors were retrospectively collected. Smoking behaviors were measured using self-reported data; thus, we cannot exclude a recollection bias, yielding an overestimation of the number of past smokers. Some past smokers may have been intermittent smokers who were not able to permanently achieve smoking cessation. Third, Hochberg multiple-group comparisons of GM volumes among the three smoking groups require the independence of tests. However, all three tests were on the same data set. Fourth, the study did not include education in adjusting GM volumes due to data limitation. Fifth, the smoking frequency and number of cigarettes may account for GM volumetric deficits, which is beyond the scope of the current study due to limited data on lifetime frequencies and doses of cigarette smoking.

In conclusion, the largest subcortical GM volumetric loss and enlargement of the lateral ventricle were observed in the past smokers among the three smoking groups, and these were more pronounced among males after adjusting GM volumes for age and intracranial volumes. Contrary to previous studies [24,25], this study found that larger subcortical region volume loss was associated with cigarette-smoking cessation, particularly among males. Our study findings contribute to a better understanding of sex differences in cigarette-smoking associations with subcortical brain region GM volumetric loss and enlargement of the lateral ventricle.

## Figures and Tables

**Table 1:** Patient characteristics by sex and smoking status.

Variables		Females (*n* = 1160)			Males (*n* = 799)		
		Active Smokers (*n* = 214)	Past Smokers (*n* = 227)	Never-Smokers (*n* = 719)	Active Smokers (*n* = 188)	Past Smokers (*n* = 224)	Never-Smokers (*n* = 387)
Age (years) ^*,+,×,∇^	Mean ± SD	49.20 ± 9.33	53.87 ± 10.40	48.91 ± 10.97	48.08 ± 9.95	53.96 ± 10.24	48.64 ± 10.19
Body Mass Index (kg/m^2^) ^*,∆,×,◊^	Mean ± SD	28.87 ± 6.19	30.67 ± 6.26	30.42 ± 6.22	27.69 ± 5.22	29.36 ± 4.43	29.14 ± 4.47
Race/ethnicity							
White	*n* (%)	61 (28.50%)	108 (47.58%)	221 (30.74%)	62 (32.98%)	103 (45.98%)	167 (43.15%)
African Americans	*n* (%)	131 (61.21%)	90 (39.65%)	371 (51.60%)	98 (52.13%)	78 (34.82%)	147 (37.98%)
Hispanics	*n* (%)	22 (10.28%)	26 (11.45%)	113 (15.72%)	23 (12.23%)	37 (16.52%)	57 (14.73%)
Other race	*n* (%)	-	3 (1.32%)	14 (1.95%)	5 (2.66%)	6 (2.68%)	16 (4.13%)
Income (USD) ^*,∆,+,×,◊^	Mean ± SD	36,926 ± 25,309	45,114 ± 27,056	49,399 ± 27,542	42,042 ± 24,881	53,871 ± 27,606	57,777 ± 28,162
Alcohol consumption (QFI) ^1,*,∆,×,◊^	Mean ± SD	126.67 ± 248.96	55.08 ± 112.35	43.61 ± 106.53	203.34 ± 313.75	136.11 ± 256.96	125.96 ± 232.58
Intracranial volume (mm^3^) ^*,+,×^	Mean ± SD	973,833 ± 138,377	1,020,970 ± 148,629	989,591 ± 148,629	1,281,107 ± 213,437	1,327,927 ± 215,539	1,312,488 ± 227,339
Smoking duration (years) ^*,×^	Mean ± SD	30.44 ± 11.69	35.16 ± 12.54	---	31.03 ± 11.45	36.48 ± 11.88	----

Notes: 1. A total of 270 participants have missing data. 2. A *p*-value < 0.05 was used for statistical significance of pairwise group comparisons; the following symbols indicate statistically significant group differences in GM volumes in each ROI stratified by sex: * female active smokers vs. female past smokers; ∆ female active smokers vs. female never-smokers; + female past smokers vs. female never-smokers; × male active smokers vs. male past smokers; ◊ male active smokers vs. male never-smokers; ∇ male past smokers vs. male never-smokers.

## Data Availability

Access to the full dataset is limited to affiliated faculty members at the university of Texas Southwestern Medical Center trained in human subject confidentiality. SAS and R programming code used in the analysis of this study and a summary data are available from the corresponding author upon reasonable request. All methods were performed in accordance with the relevant guidelines and regulations.

## Supplementary Materials

The following supporting information can be downloaded at https://www.mdpi.com/article/10.3390/brainsci13081164/s1. Figure S1: Pairwise comparison of age-, intracranial volume-, and annual alcohol consumption adjusted GM volumes using only subjects with complete data on alcohol consumption stratified by sex. Table S1 Mean and standard deviation (mean ± SD) of unadjusted subcortical region volumes (mm^3^) by gender and smoking status. Table S2 Mean and standard deviations (mean ± SD) of age-, intracranial volume-adjusted subcortical region volumes by gender and smoking status. Table S3 Means and standard deviations (mean ± SD) of age-, intracranial volume-, and annual alcohol consumption adjusted subcortical region volumes by gender and smoking status. Table S4 Means and standard deviations (mean ± SD) of age-, intracranial volume-, and smoking duration adjusted subcortical region volumes between active and past smoker by gender.
