# Correction: Chen et al. Sex Difference in Cigarette-Smoking Status and Its Association with Brain Volumes Using Large-Scale Community-Representative Data. *Brain Sci.* 2023, *13*, 1164

**DOI:** 10.3390/brainsci15070687

**Published:** 2025-06-26

**Authors:** Xiaofei Chen, Riley Cook, Francesca M. Filbey, Hang Nguyen, Roderick McColl, Haekyung Jeon-Slaughter

**Affiliations:** 1 Department of Statistics and Data Science, Southern Methodist University, Dallas, TX 75205, USA; xiaofeic@mail.smu.edu (X.C.); hangnguyen@mail.smu.edu (H.N.); 2 VA North Texas Health Care Service, Dallas, TX 75216, USA; riley.cook@va.gov; 3 School of Behavioral and Brain Sciences, University of Texas at Dallas, Richardson, TX 75080, USA; francesca.filbey@utdallas.edu; 4 Department of Radiology, University of Texas Southwestern Medical Center, Dallas, TX 75390, USA; roderick.mccoll@utsouthwestern.edu; 5 Department of Internal Medicine, University of Texas Southwestern Medical Center, Dallas, TX 75390, USA

## Error in Table

In the original publication [1], there was a mistake in Table 1. Patient characteristics by sex and smoking status, as published. The unit of Intracranial Volume was incorrect. The corrected unit is “Intracranial volume (mm^3^)”. There was also a mistake in the title of Table S1, as published. The corrected title is “Mean and standard deviation (mean ± SD) of unadjusted subcortical region volumes (mm^3^) by gender and smoking status”. The authors state that the scientific conclusions are unaffected. This correction was approved by the Academic Editor. The original publication has also been updated.
