# Supplementary material for: Sex Difference in Cigarette-Smoking Status and Its Association with Brain Volumes Using Large-Scale Community-Representative Data
Source: Brain Sci. 2023 Aug 4;13(8):1164. doi: 10.3390/brainsci13081164 (PMC10452722; doi:10.3390/brainsci13081164)
Supplement: Supplementary file 1 [file brainsci-13-01164-s001.zip › brainsci-2506305-supplementary.pdf]

Supplementary materials

"Sex differential in cigarette smoking status and its association with brain volumes using a large-scale community representative data"

Chen, Cook, Filbey, Nguyen, McColl & Jeon-Slaughter

Table S1. Mean and standard deviation (mean ± SD) of unadjusted subcortical region volumes (mm^3) by gender and smoking status

		Females			Males		
		Active smokers	Past smokers	Never smokers	Active smokers	Past smokers	Never smokers
Regions	Volumes	mean±SD	mean±SD	mean±SD	mean±SD	mean±SD	mean±SD
Amygdala	Total$^{\Delta,+,\lozenge}$	2895.4 ±406.2	2887.2 ±409.4	2998.0 ±364.5	3117.5 ±405.3	3175.1 ±416.0	3210.8 ±422.9
	Right$^{\Delta,+,\lozenge}$	1512.7 ±229.8	1508.8 ±226.6	1572.9 ±207.7	1617.9 ±216.7	1647.6 ±233.0	1672.9 ±230.7
	Left$^{\Delta,+,\lozenge}$	1382.7 ±203.8	1378.5 ±205.7	1425.1 ±186.1	1499.6 ±211.0	1527.6 ±214.8	1537.9 ±217.8
Insula	Total$^{\Delta,+,\lozenge,\nabla}$	9483.3 ±1040.0	9551.31 ±1088.22	9723.7 ±1040.0	10480.7 ±1222.2	10535.4 ±1137.1	10935.7 ±1222.1
	Right$^{\Delta,\lozenge,\nabla}$	4748.8 ±535.4	4796.4 ±565.2	4879.0 ±557.1	5278.7 ±640.8	5288.2 ±597.0	5514.7 ±630.8
	Left$^{\Delta,+,\lozenge,\nabla}$	4734.4 ±537.7	4754.9 ±559.1	4844.8 ±546.3	5202.0 ±625.5	5247.2 ±580.6	5421.0 ±628.5
Lateral Ventricle	Total$^{*,+,\times,\lozenge}$	11543.4 ±6574.5	15161.5 ±10330.0	12560.1 ±7703.5	15391.6 ±7799.3	19429.3 ±11683.7	17678.5 ±11680.9
	Right$^{*,+,\times,\lozenge}$	5643.4 ±3300.6	7266.2 ±4936.5	5997.2 ±3690.3	7379.7 ±3817.3	9384.5 ±5704.5	8515.9 ±5843.3
	Left$^{*,\Delta,+,\times,\lozenge}$	5900.0 ±3475.8	7895.3 ±5558.8	6563.0 ±4190.0	8011.8 ±4306.1	10044.8 ±6245.1	9126.6 ±6174.9
Pallidum	Total	2966.2 ±366.4	2927.9 ±412.51	2983.2 ±392.2	3376.5 ±542.1	3293.0 ±508.1	3366.1 ±499.1
	Right	1411.7 ±194.6	1399.6 ±217.1	1425.6 ±219.0	1622.7 ±302.1	1578.8 ±278.7	1614.3 ±268.9
	Left	1554.6 ±198.8	1528.3 ±230.4	1557.7 ±206.6	1753.8 ±274.1	1714.2 ±261.5	1751.8 ±263.6
Hippacampus	Total$^{+,\lozenge}$	7702.0 ±857.7	7606.2 ±962.1	7791.7 ±746.95	8223.3 ±841.8	8325.3 ±955.6	8405.5 ±980.8
	Right$^{\Delta,+,\lozenge}$	3833.0 ±437.0	3794.9 ±483.5	3899.7 ±391.0	4093.3 ±430.4	4139.8 ±482.4	4181.3 ±498.8
	Left$^{+,\lozenge}$	3868.9 ±440.5	3811.3 ±499.2	3892.0 ±386.6	4130.1 ±439.3	4185.5 ±497.7	4224.2 ±510.8
Caudate	Total	6080.1 ±868.9	6086.6 ±909.8	6074.4 ±849.7	6704.4 ±919.4	6698.1 ±937.4	6852.6 ±1049.7

	Right$^{\nabla}$	3077.5 ±445.3	3087.6 ±463.2	3078.6 ±442.2	3391.7 ±476.4	3380.9 ±492.4	3468.2 ±542.2
	Left	3002.6 ±442.9	2999.0 ±463.5	2995.8 ±428.2	3312.7 ±466.5	3317.3 ±468.3	3384.4 ±527.4
Putamen	Total	9480.0 ±1073.3	9406.2 ±1095.8	9484.5 ±1101.4	10394.6 ±1290.0	10311.0 ±1261.2	10495.6 ±1297.4
	Right	4667.4 ±540.9	4630.5 ±557.6	4654.0 ±554.5	5135.5 ±637.8	5106.3 ±654.5	5190.2 ±666.0
	Left	4812.6 ±562.1	4775.7 ±582.3	4830.5 ±577.3	5259.1 ±687.8	5204.7 ±651.92	5305.5 ±665.89
Accumbens area	Total$^{+,\nabla}$	1069.7 ±178.7	1051.9 ±168.4	1093.4 ±174.2	1157.7 ±188.8	1125.0 ±189.0	1173.7 ±185.6
	Right$^{\Delta,+,\nabla}$	568.1 ±101.2	560.1 ±96.4	583.6 ±98.6	610.5 ±109.34	595.3 ±109.0	623.9 ±108.0
	Left$^{+,\nabla}$	501.6 ±100.00	491.8 ±100.8	509.9 ±101.1	547.2 ±114.2	529.8 ±104.8	549.9 ±101.3
Thalamus	Total^{+}	12097.1 ±1330.7	12072.7 ±1504.7	12300.4 ±1359.1	13620.3 ±1637.0	13680.9 ±1836.1	13865.1 ±1716.6
	Right$^{\Delta,+}$	5931.1 ±624.7	5910.9 ±725.3	6037.5 ±670.7	6562.6 ±742.1	6570.1 ±829.4	6659.1 ±791.1
	Left	6166.1 ±736.4	6161.8 ±812.6	6262.9 ±723.1	7057.7 ±940.0	7110.8 ±1064.2	7205.9 ±974.9

Notes. 1. *P*-value < 0.05 for pairwise group comparisons; The following symbols present significant smoking group differences per pairwise stratified by gender ($p < 0.05$). 2. *P*-value < 0.05 for post hoc group comparisons; *Female current smokers vs. Female past smokers; Δ Female current smokers vs. Female never smokers; + Female past smokers vs. Female never smokers; × Male current smokers vs. Male past smokers; ◊ Male current smokers vs. Male never smokers; ∇ Male past smokers vs. Male never smokers

Tables S2. Mean and standard deviations (mean ± SD) of age-, intracranial volume-adjusted subcortical region volumes by gender and smoking status

		Females			Males		
		Active smokers	Past smokers	Never smokers	Active smokers	Past smokers	Never smokers
Regions	Volumes	mean±SD	mean±SD	mean±SD	mean±SD	mean±SD	mean±SD
Amygdala	Total$^{+,\times,\nabla}$	2,952.93 ± 96.50	2,941.94 ± 110.28	2,963.61 ± 110.58	3,192.76 ± 135.73	3,138.43 ± 131.88	3,195.49 ± 135.25
	Right$^{+,\times,\nabla}$	1,547.41 ± 46.08	1,540.97 ± 52.59	1,552.39 ± 53.02	1,661.06 ± 68.92	1,630.91 ± 67.46	1,661.61 ± 68.78
	Left$^{+,\times,\nabla}$	1,405.52 ± 50.51	1,400.98 ± 57.79	1,411.22 ± 57.66	1,531.70 ± 67.20	1,507.52 ± 64.88	1,533.88 ± 66.94
Insula	Total$^{\times,\nabla}$	9,607.95 ± 517.38	9,605.64 ± 593.90	9,669.48 ± 582.25	10,727.57 ± 584.65	10,613.69 ± 549.36	10,770.41 ± 577.59
	Right$^{\times,\nabla}$	4,819.57 ± 254.99	4,821.37 ± 292.81	4,850.05 ± 286.40	5,400.79 ± 290.56	5,344.57 ± 277.79	5,422.77 ± 292.19
	Left$^{\times,\nabla}$	4,788.38 ± 262.49	4,784.27 ± 301.20	4,819.43 ± 295.96	5,326.78 ± 284.10	5,269.12 ± 271.59	5,347.64 ± 285.43
Lateral Ventric	Total$^{*,+,\times,\nabla}$	12,194 ± 4,579.24	14,538 ± 5,174.51	12,563 ± 4,976.15	16,330 ± 5,494.94	19,491 ± 5,977.70	17,186 ± 6,035.78
	Right$^{*,+,\times,\nabla}$	5,856.70 ±2,155.59	6,964.87 ±2,435.07	6,028.82 ±2,343.83	7,867.28 ±2,649.05	9,379.50 ±2,880.10	8,281.89 ±2,910.56
	Left$^{*,+,\times,\nabla}$	6,337.29 ±2,423.81	7,573.13 ±2,739.61	6,534.52 ±2,632.52	8,462.74 ±2,846.43	10,112.34 ±3,098.06	8,904.48 ±3,125.71
Pallidum	Total$^{+,\times,\nabla}$	2,963.70 ± 168.77	2,935.88 ± 192.36	2,981.48 ± 194.91	3,368.34 ± 272.21	3,277.27 ± 262.01	3,379.14 ± 271.27
	Right$^{+,\times,\nabla}$	1,415.08 ± 87.64	1,400.50 ± 99.54	1,424.27 ± 100.90	1,617.90 ± 137.01	1,568.77 ± 132.26	1,622.39 ± 136.48
	Left$^{+,\times,\nabla}$	1,548.61 ± 81.43	1,535.38 ± 92.83	1,557.22 ± 94.01	1,750.44 ± 135.47	1,708.50 ± 130.07	1,756.75 ± 135.11
Hippocampus	Total$^{\times,\nabla}$	7,716.46 ± 268.51	7,727.92 ± 308.61	7,748.97 ± 299.76	8,366.68 ± 376.10	8,244.60 ± 361.62	8,382.64 ± 374.90
	Right$^{\times,\nabla}$	3,855.85 ± 133.66	3,861.10 ± 153.62	3,872.02 ± 149.31	5,163.51 ± 184.70	4,099.88 ± 177.98	4,170.31 ± 184.02
	Left$^{\times,\nabla}$	3,860.61 ± 134.85	3,866.82 ± 155.00	3,876.95 ± 150.46	4,203.17 ± 191.63	4,144.71 ± 183.92	4,212.33 ± 191.15
Caudate	Total	6,035.32 ± 395.22	6,098.66 ± 454.87	6083.90 ± 432.95	6,757.27 ± 473.48	6,732.28 ± 456.57	6,807.12 ± 484.21
	Right	3,057.66 ± 204.85	3,093.00 ± 235.76	3,082.81 ± 223.99	3,416.79 ± 242.18	3,404.43 ± 233.57	3,442.37 ± 247.72
	Left	2,977.67 ± 190.48	3,005.66 ± 219.23	3,001.09 ± 209.08	3,340.49 ± 231.31	3,327.85 ± 223.01	3,364.76 ± 236.49
Putamen	Total$^{+,\times,\nabla}$	9,455.64 ± 491.54	9,361.47 ± 559.39	9,505.84 ± 569.87	10,462.38 ± 710.62	10,250.57 ± 681.65	10,49767 ± 709.09
	Right$^{+,\times,\nabla}$	4,644.83 ± 232.59	4,604.10 ± 264.95	4,669.06 ± 269.02	5,173.99 ± 343.06	5,072.29 ± 329.03	5,191.17 ± 342.35

	Left[*,+,×,∇]	4,810.81 ± 259.16	4,757.37 ± 294.67	4,836.78 ± 301.05	5,288.39 ± 367.56	5,178.28 ± 352.62	5,306.49 ± 366.74
Accumbens area	Total[+,×,∇]	1,079.51 ± 55.47	1,068.79 ± 63.12	1,085.16 ± 64.32	1,162.38 ± 70.99	1,136.78 ± 68.55	1,164.67 ± 70.72
	Right[+,×,∇]	575.35 ± 26.36	570.62 ± 30.02	578.09 ± 30.51	616.96 ± 36.64	601.11 ± 35.83	617.32 ± 36.55
	Left[*,+,×,∇]	504.15 ± 29.12	498.17 ± 33.11	507.08 ± 33.83	545.42 ± 35.08	535.67 ± 33.60	547.35 ± 35.04
Thalamus	Total[+,×,∇]	12,172 ± 758.57	12,128 ± 869.10	12,260 ± 861.36	13,791.03 ±1,047.16	13,546.07 ±1,001.32	13,860.18 ±1,049.22
	Right[+,×,∇]	5,973.62 ± 354.35	5,945.28 ± 405.61	6,013.93 ± 403.85	6,633.00 ± 451.82	6,511.49 ± 432.59	6,658.88 ± 451.57
	Left[×,∇]	6,198.96 ± 404.73	6,183.55 ± 464.06	6,246.27 ± 458.07	7,158.03 ± 596.68	7,034.58 ± 7570.40	7,201.30 ± 599.25

Notes. 1. *P*-value < 0.05 for pairwise group comparisons; The following symbols present significant smoking group differences per pairwise stratified by gender ($p < 0.05$). 2. *P*-value < 0.05 for post hoc group comparisons; *Female current smokers vs. Female past smokers; Δ Female current smokers vs. Female never smokers; + Female past smokers vs. Female never smokers; × Male current smokers vs. Male past smokers; ◊ Male current smokers vs. Male never smokers; ∇ Male past smokers vs. Male never smokers

Table S3. Means and standard deviations (mean ± SD) of age-, intracranial volume-, and annual alcohol consumption adjusted subcortical region volumes by gender and smoking status

		Females			Males		
		Active smokers	Past smokers	Never smokers	Active smokers	Past smokers	Never smokers
Regions	Volumes	mean±SD	mean±SD	mean±SD	mean±SD	mean±SD	mean±SD
Amygdala	Total[Δ,+,×,∇]	2943.7 ±101.3	2947.0 ±104.0	2972.6 ±102.0	3195.3 ±144.3	3138.9 ±139.9	3204.5 ±142.9
	Right[Δ,+,×,∇]	1543.2 ±48.4	1544.6 ±48.0	1557.7 ±47.4	1662.1 ±74.2	1631.5 ±72.2	1667.3 ±73.3
	Left[Δ,+,×,∇]	1400.5 ±53.2	1402.4 ±56.2	1414.9 ±54.8	1533.2 ±70.7	1507.4 ±68.4	1537.3 ±70.2
Insula	Total[Δ,×,∇]	9583.9 ±518.6	9619.1 ±587.7	9701.2 ±561.8	10690.5 ±582.1	10608.4 ±558.8	10761.6 ±572.8
	Right[Δ,×,∇]	4807.8 ±254.8	4826.9 ±289.9	4864.7 ±276.6	5379.3 ±294.7	5342.2 ±282.7	5417.7 ±289.1
	Left[Δ,×,∇]	4776.1 ±263.9	4792.2 ±297.8	4836.5 ±285.3	5311.2 ±287.7	5266.2 ±276.3	5343.9 ±283.9
Lateral Ventricle	Total[*,+,×,∇]	12124.3 ±4661.3	14975.0 ±5523.9	12716.2 ±5280.5	16284.6 ±5432.0	19822.7 ±5913.6	17047.3 ±5945.6
	Right[*,+,×,∇]	5850.3 ±2195.5	7189.2 ±2601.6	6115.1 ±2490.6	7867.9 ±2612.8	9550.2 ±2843.5	8225.3 ±2860.9
	Left[*,+,×,∇]	6274.0 ±2466.5	7785.9 ±2922.5	6601.1 ±2790.2	8416.7 ±2820.4	10272.6 ±3070.8	8822.0 ±3085.4
Pallidum	Total[Δ,+,×,∇]	2953.5 ±168.2	2936.6 ±179.0	2993.1 ±179.1	3357.1 ±264.4	3277.4 ±254.3	3379.8 ±260.5
	Right[Δ,+,×,∇]	1408.4 ±87.7	1397.3 ±93.4	1428.4 ±93.9	1610.5 ±131.8	1567.0 ±127.0	1621.3 ±129.8
	Left[Δ,+,×,∇]	1545.1 ±80.7	1539.3 ±85.8	1564.7 ±85.4	1746.5 ±133.0	1710.4 ±127.7	1758.5 ±131.1
Hippocampus	Total[Δ,×,∇]	7724.2 ±260.8	7750.6 ±298.2	7782.8 ±283.0	8352.2 ±411.7	8236.2 ±395.1	8393.7 ±403.1
	Right[Δ,×,∇]	3855.9 ±130.5	3869.9 ±147.3	3887.3 ±140.1	4154.2 ±204.8	4095.9 ±196.3	4176.2 ±199.5
	Left[Δ,×,∇]	3868.3 ±130.7	3880.7 ±151.0	3895.4 ±143.1	4198.0 ±207.5	4140.4 ±199.3	4217.5 ±204.0
Caudate	Total[*,Δ]	6022.0 ±382.4	6117.4 ±451.7	6103.1 ±419.1	6735.5 ±467.4	6740.4 ±454.6	6801.8 ±466.5
	Right[*,Δ]	3049.6 ±197.1	3101.2 ±233.6	2090.6 ±216.5	3405.6 ±238.6	3408.9 ±231.9	3440.2 ±237.7
	Left[*,Δ]	2972.3 ±185.5	3016.2 ±218.3	3012.5 ±202.8	3329.9 ±228.9	3331.5 ±222.7	3361.5 ±228.8
Putamen	Total[Δ,+,×,∇]	9425.9 ±490.6	9357.2 ±529.3	9530.3 ±531.5	10450.9 ±709.3	10261.1 ±681.4	10514.2 ±699.5
	Right[Δ,+,×,∇]	4628.5 ±233.0	4603.0 ±250.1	4681.3 ±250.1	5163.8 ±338.7	5078.0 ±325.0	5197.2 ±333.0

	Left[Δ,*,+,×,∇]	4797.4 ±258.0	4754.2 ±279.5	4849.0 ±281.6	5287.1 ±371.1	5183.1 ±356.7	5317.0 ±366.8
Accumbens area	Total[Δ,+,×,∇]	1080.1 ±55.6	1070.5 ±60.9	1090.4 ±61.2	1162.7 ±69.1	1138.6 ±66.7	1166.5 ±68.6
	Right[Δ,+,×,∇]	574.7 ±27.0	571.4 ±28.8	580.8 ±28.9	616.6 ±34.6	601.8 ±33.7	618.2 ±34.4
	Left[*,+,×,∇]	505.3 ±29.1	499.1 ±32.2	509.6 ±32.3	546.0 ±35.4	536.7 ±34.0	548.3 ±35.2
Thalamus	Total[Δ,+,×,∇]	12143.9 ±773.3	12148.2 ±860.7	12320.2 ±834.7	13745.5 ±1041.8	13535.2 ±999.6	13856.1 ±1026.0
	Right[Δ,+,×,∇]	5957.6 ±361.6	5947.4 ±492.2	6936.6 ±392.3	6615.4 ±455.0	6508.9 ±436.6	6661.5 ±447.7
	Left[Δ,×,∇]	6186.3 ±214.5	6200.8 ±459.2	6283.6 ±443.1	7130.0 ±587.7	7026.3 ±564.2	7194.7 ±579.6

Notes. 1. 270 observations are not included due to missing data on annual alcohol consumption.

2. *P*-value < 0.05 for pairwise group comparisons; *Female current smokers vs. Female past smokers; Δ Female current smokers vs. Female never smokers; + Female past smokers vs. Female never smokers; × Male current smokers vs. Male past smokers; ◊ Male current smokers vs. Male never smokers; ∇ Male past smokers vs. Male never smokers

Table S4. Means and standard deviations (mean ± SD) of age-, intracranial volume-, and smoking duration adjusted subcortical region volumes between active and past smoker by gender

		Females			Males		
**Regions**	Volumes (mL)	Active (*n*=214)	Past (*n*=227)	*P* value[1]	Active (*n*=188)	Past (n=224)	*P* value[1]
**Amygdala**	Total	2915.8 ±105.2	2900.1 ±116.5	0.1369	3185.2 ±136.9	3130.1 ±133.1	<0.0001
	Left	1389.3 ±54.1561	1382.6 ±60.2965	0.2249	1529.6 ±67.4960	1505.2 ±65.2069	0.0002
	Right	1526.6 ±51.2	1517.4 ±56.4	0.0761	1655.6 ±69.8	1624.9 ±68.4	<0.0001
**Insula**	Total	9517.3 ±534.8	9503.2 ±603.9	0.7964	10617.1 ±588.1	10493.0 ±564.7	0.0299
	Left	4743.7 ±271.2	4733.8 ±306.3	0.7202	5280.8 ±289.6	5218.9 ±277.9	0.0277
	Right	4773.5 ±263.7	4769.4 ±297.7	0.8770	5336.2 ±298.5	5274.1 ±286.9	0.0321
**Lateral Ventric**	Total	12265.8 ±4583.1	14619.1 ±5184.8	<0.0001	15935.6 ±5450.4	19061.1 ±5939.8	<0.0001
	Left	6352.7 ±2424.6	7590.6 ±2741.8	<0.0001	8248.2 ±2821.9	9878.0 ±3077.1	<0.0001
	Right	5913.1 ±2158.8	7028.6 ±2443.3	<0.0001	7687.5 ±2629.0	9183.1 ±2863.0	<0.0001
**Pallidum**	Total	2967.0 ±168.1	2939.6 ±191.9	0.1124	3373.3 ±271.6	3282.7 ±261.3	0.0006
	Left	1549.8 ±81.1	1536.7 ±92.6	0.1162	1753.3 ±135.1	1711.6 ±129.7	0.0015
	Right	1417.2 ±86.9	1402.9 ± 99.2	0.1089	1620.0 ±136.7	1571.1 ±132.0	0.0003
**Hippocampus**	Total	7667.1 ±277.4	7672.2 ±313.3	0.8576	8358.6 ±377.2	8235.7 ±362.8	0.0008
	Left	3843.0 ±137.9	3846.9 ±156.5	0.7801	4200.0 ±192.0	4141.3 ±184.4	0.0017
	Right	3824.1 ±139.6	3825.2 ±156.8	0.9361	4158.5 ±185.4	4094.4 ±178.7	0.0004
**Caudate**	Total	6059.8 ±392.3	6126.3 ±454.3	0.1015	6744.5 ±474.4	6718.3 ±457.7	0.5700
	Left	2991.8 ±188.8	3021.7 ±218.9	0.1271	3337.7 ±231.5	3324.8 ±223.2	0.5662
	Right	3067.9 ±203.7	3104.6 ±235.6	0.0818	3406.8 ±242.9	3393.5 ±234.4	0.5736
**Putamen**	Total	9504.6 ±481.7	9416.8 ±552.4	0.0766	10487.2 ±707.6	10277.7 ±678.3	0.0023
	Left	4827.3 ±255.7	4776.0 ±292.2	0.0509	5299.1 ±366.2	5189.9 ±351.2	0.0022

	Right	4677.3 ±226.3	4640.8 ±260.7	0.1178	5188.1 ±341.3	5087.7 ±327.1	0.0025
**Accumbens area**	Total	1071.7 ±57.2	1060.0 ±64.4	0.0443	1158.7 ±71.5	1132.7 ±69.12	0.0002
	Left	501.5 ±29.7	495.2 ±33.5	0.0370	544.2 ±35.2	534.3 ±33.7	0.0040
	Right	570.2 ±27.54	564.8 ±30.9	0.0538	614.5 ±37.0	598.4 ±36.2	<0.0001
**Thalamus**	Total	12110.2 ±770.7	12058.4 ±876.5	0.5109	13773.3 ±1049.2	13526.7 ±1003.6	0.0154
	Left	6171.4 ±409.9	6152.5 ±467.1	0.6512	7147.2 ±597.8	7022.8 ±571.7	0.0317
	Right	5938.7 ±361.4	5905.9 ±410.1	0.3736	6626.1 ±452.7	6503.9 ±433.5	0.0055

Note. 1. 2-sided T tests

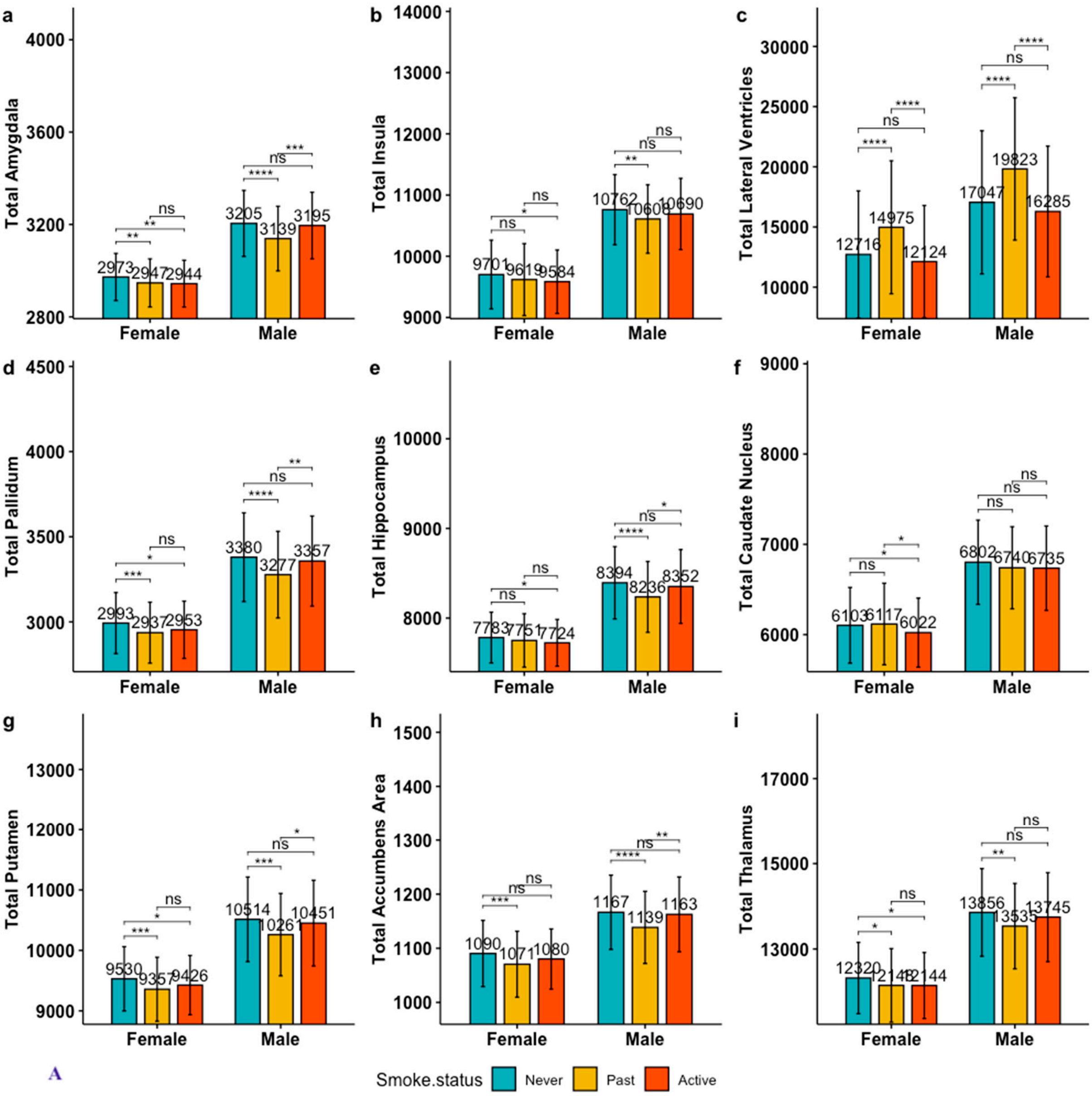
a
Total Amygdala
b
Total Insula
c
Total Lateral Ventricles
d
Total Pallidum
e
Total Hippocampus
f
Total Caudate Nucleus
g
Total Putamen
h
Total Accumbens Area
i
Total Thalamus
Female
Male
A
Smoke.status
Never
Past
Active

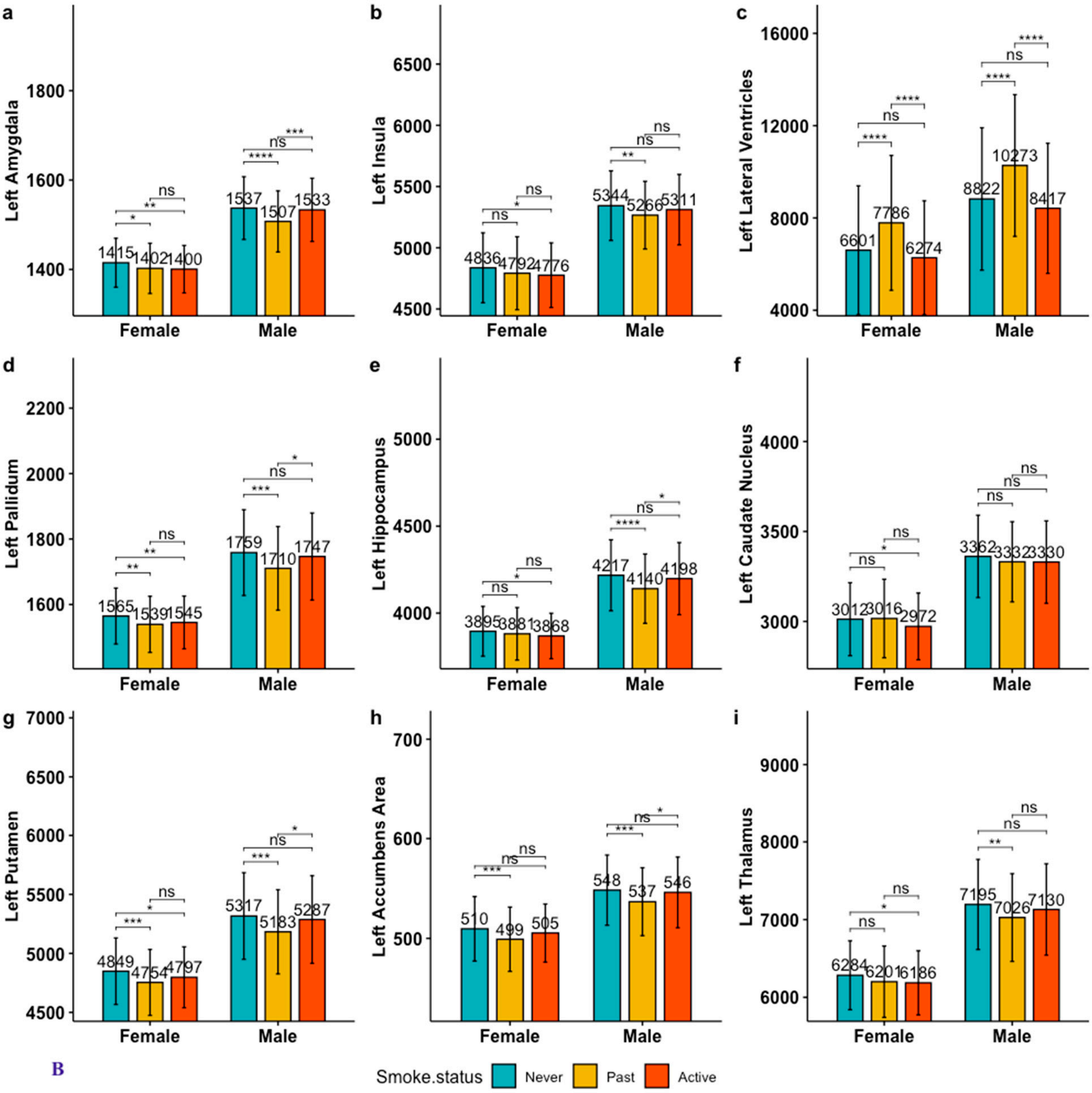

a
Left Amygdala
1400
1600
1800
1415
1402
1400
1537
1507
1533
Female
Male
b
Left Insula
4500
5000
5500
6000
6500
4836
4792
4776
5344
5266
5311
c
Left Lateral Ventricles
4000
8000
12000
16000
6601
7786
6274
8822
10273
8417
d
Left Pallidum
1600
1800
2000
2200
1565
1539
1545
1759
1710
1747
e
Left Hippocampus
4000
4500
5000
3895
3881
3868
4217
4140
4198
f
Left Caudate Nucleus
3000
3500
4000
3012
3016
2972
3362
3332
3330
g
Left Putamen
4500
5000
5500
6000
6500
7000
4849
4754
4797
5317
5183
5287
h
Left Accumbens Area
500
600
700
510
499
505
548
537
546
i
Left Thalamus
6000
7000
8000
9000
6284
6201
6186
7195
7026
7130
B
Smoke.status
Never
Past
Active

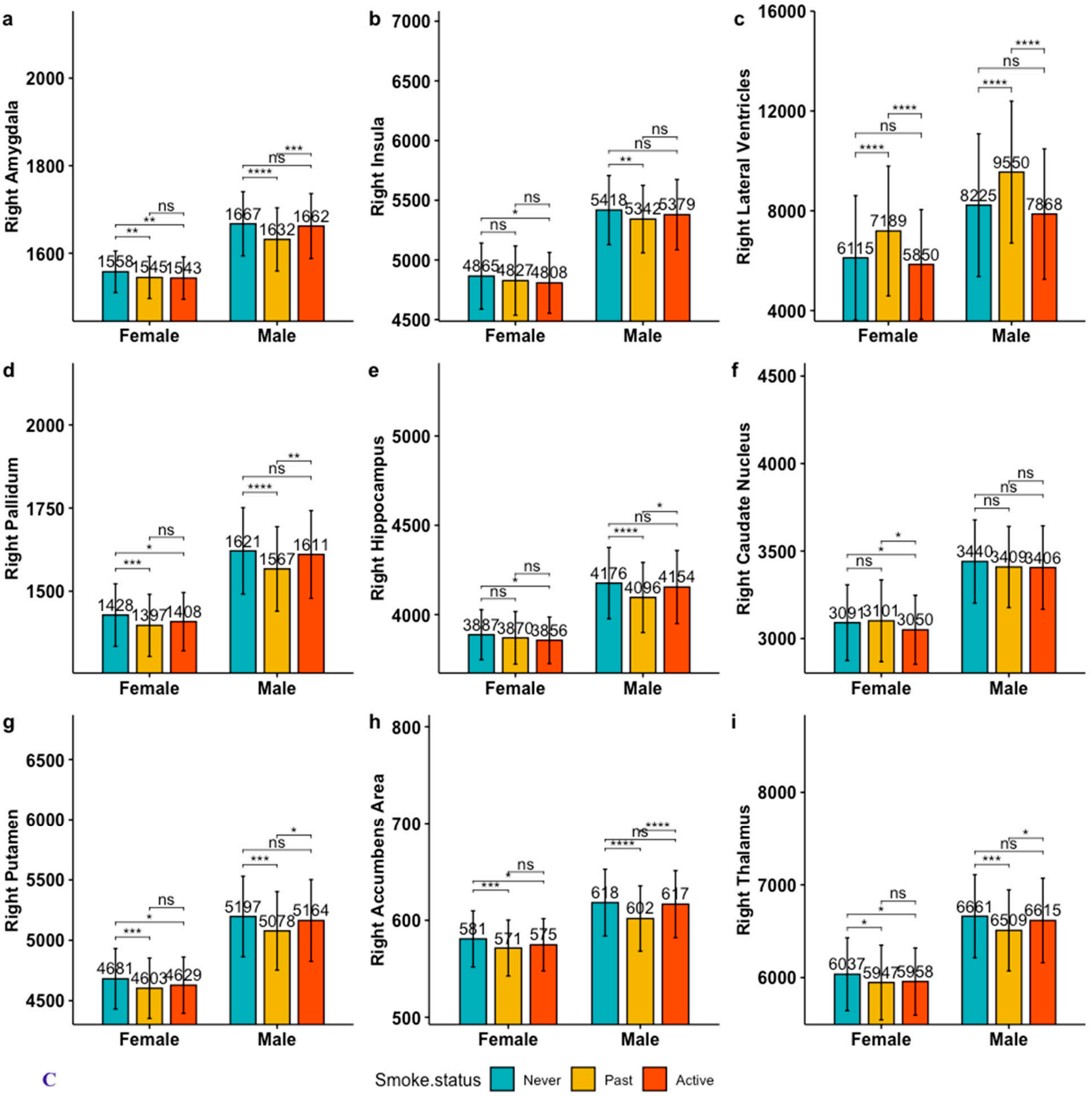

Figure S1. Pairwise comparison of age -, intracranial volume-, and annual alcohol consumption adjusted GM volumes using only subjects with complete data on alcohol consumption stratified by sex**. Notes.** 1. Panel S1.A. represents Total GM volumes, Panel S1.B. Left side volumes, and Panel S1.C. Right side volumes; 2. GM volumes were compared by pairwise smoking groups, never vs past smokers; never vs active smokers; and past and active smokers. Hochberg correction was used for multiple group comparison statistical significance at α=0.05; 3. ****: <0.0001; ***: <0.001; **: <0.01; *: <0.05; "ns": not significant.
